# Lung function impairment and cardiometabolic risks among rural adults: implication for an aging society

**DOI:** 10.1186/s12889-021-10990-8

**Published:** 2021-05-20

**Authors:** Yu-Chih Lin, Tung-Jung Huang, Mei-Hua Yeh, Ming-Shyan Lin, Mei-Yen Chen

**Affiliations:** 1 Department of Family Medicine, Chang Gung Memorial Hospital, Chiayi, Yunlin Taiwan; 2 Department of Internal Medicine, Chang Gung Memorial Hospital, Chiayi, Yunlin Taiwan; 3 Department of Respiratory Care, Chang Gung University of Science and Technology, Chiayi, Taiwan; 4 Department of Respiratory Therapy, Chang Gung Memorial Hospital, Chiayi, Yunlin Taiwan; 5 Department of Cardiology, Chang Gung Memorial Hospital, No. 2, Chiapu Rd. West Sec, Putz City, 61363 Chiayi Taiwan, ROC; 6 Department of Nursing, Chang Gung University of Science and Technology, Chiayi, Taiwan; 7 School of Nursing, Chang Gung University, Taoyuan, Taiwan

**Keywords:** Impaired lung function, Cardiometabolic risks, Metabolic syndrome, Healthy lifestyle, Rural

## Abstract

**Background:**

Early detection and prevention of cardiometabolic risk factors in an increasingly aging society are a global public health concern. Maintaining adequate lung function is important for healthy aging. Few studies exist on lung function impairment and decline in primary healthcare settings, especially among rural adults with cardiometabolic risks. This study aimed to explore the prevalence of impaired lung function and its association with cardiometabolic risks among rural adults.

**Methods:**

A community-based, cross-sectional study was conducted between March and December 2019 in western coastal Yunlin County, Taiwan. The lung function test was measured by spirometry, based on the American Thoracic Society recommendations. Three lung function parameters were obstructive lung impairment, restrictive lung impairment, and mixed lung impairment. Restrictive, obstructive, and mixed type lung function was categorized as impaired. Cardiometabolic risk factors and metabolic syndrome were based on the national standard and include five abnormal biomarkers, including abdominal obesity, blood pressure, fasting plasma glucose, triglycerides, and decreased high-density cholesterol levels.

**Results:**

The median age of the 1653 (92.9%) participants with complete data was 66years (interquartile range: 55 to 75years). The prevalence of impaired lung function was 37%, including 31.7% restrictive, 2.5% obstructive, and 2.7% mixed type. Adults with impaired lung function (86% restrictive type) engaged more in smoking and betel nut chewing, ate fewer vegetables and fruit, and drank less water compared to the normal lung function group. After adjusting for potential confounding variables, multivariate logistic regression analysis showed that cardiometabolic risk factors were independently associated with restrictive lung impairment, while cigarette smoking (OR=2.27, 95% CI=1.144.53) and betel nut chewing (OR=2.33, 95% CI=1.095.01) were significantly associated with the obstructive type of lung impairment.

**Conclusions:**

A high prevalence of restrictive lung impairment, cardiometabolic risks, and unhealthy lifestyles among rural adults were found in this study. For adults with cardiometabolic risks in rural areas, initiating lifestyle modifications with culture-tailored programs to improve lung function should be an important issue for clinicians and primary healthcare providers.

## Background

With the increase in the aging population, older individuals are more likely to develop chronic conditions, such as cardiovascular diseases, stroke, diabetes mellitus, and chronic obstructive pulmonary disease [1, 2]. This phenomenon not only causes fiscal burden but also affects the sustenance of the Taiwan National Health Insurance. To address this trend, the Taiwanese government launched a new care paradigm, Long-term Care 2.0, for preventing and delaying disability by promoting healthy aging through exercise, providing good nutrition, and encouraging social participation in the community activity centers [3]. As the burden of cardiovascular disease and diabetes mellitus remains tremendous, risk factors leading to these diseases were intensively studied in the past decades. The term cardiometabolic risk was first employed by the American Diabetes Association as an umbrella term to include all the risk factors for diabetes and cardiovascular diseases [4,7]. Cardiometabolic risk factors refer to the combined factors contributing to cardiovascular events and the interrelated pathophysiology of metabolic disorders. Several cardiometabolic risk factors were proposed, including age, sex, hypertension, dyslipidemia, hyperglycemia, abdominal obesity (measured by the waist circumference), insulin resistance, inflammation, cigarette smoking, betel nut chewing, alcohol drinking, lack of fruits/vegetable consumption, and sedentary lifestyle [4,7]. These factors are associated with vascular events or type 2 diabetes [8]. The pathophysiology of abdominal fat and insulin resistance contributes significantly to increased cardiometabolic risk [4, 9].

Metabolic syndrome (MetS) is defined as a constellation of at least three out of five cardiometabolic risk factors, including abdominal obesity, elevated blood pressure, fasting plasma glucose, triglycerides (TG), and decreased high-density cholesterol (HDL-C) levels [1, 9]. Besides smoking, these factors are recognized indicators to predict cardiovascular events, including coronary heart disease, heart failure, stroke, and hypertension [10] or metabolic disorders (such as diabetes mellitus or dyslipidemia). The five leading modifiable risk factors (hypercholesterolemia, diabetes, hypertension, obesity, and smoking) are reported to be responsible for more than half of cardiovascular mortality [11]. Further, the International Diabetes Federation revealed that, besides smoking, MetS is a cluster of the most high-risk factors for cardiovascular accidents [9]. A systematic review reported that the prevalence of MetS is increasing among adults in the Asia-pacific region [12]. Recently, some studies indicated that diabetes and poor glycemic control is associated with lung function impairment, especially of the restrictive type [13,15]. Besides, low pulmonary function is related to a high risk of low muscle mass and sarcopenia in healthy community-dwelling older adults [16]. Some studies indicated the benefits of physical activity and a healthy diet for adults with cardiometabolic diseases, and active adults have better lung function and slower age-related decline [17, 18].

At rest, humans take 1215 breaths per minute, each breath contains a maximum of 500mL of air, and the lung inspires and expires around 68L of air per minute [19]. The maximal lung function capacity occurs around age 20years in females and 25years in males. This starts declining after 35years of age due to the loss of lung elasticity, weakened muscles of respiration, and decreased surface area for alveolar gas exchange [19,22]. The lung function can be assessed by using a spirometer to measure the air volume during inspiration and/or expiration. The clinicians frequently use three indices to identify airway diseases: (1) forced expiratory vital capacity (FVC) refers to the total amount of air that an individual can exhale in one breath, (2) the predicted FVC value (%), and (3) the forced expiratory volume in one second (FEV1)/FVC ratio (%) [18]. Many factors, including age, body mass index, sex, ethnicity, physical activity, environmental conditions, altitude, smoking, and socioeconomic status, influence the lung function values [19, 20, 23]. Many developed countries have initiated health strategies to reduce the health impact on the aging society. However, few studies have focused on the association between impaired lung function and cardiometabolic risks among adults in rural areas of Taiwan. This study aimed to explore the prevalence of impaired lung function and its association with cardiometabolic risks among rural adults.

## Methods

### Design, sample, and setting

A community-based, cross-sectional study design was applied. This study is the first phase of a nurse-led community health development program to prevent and slow down age-related disability among rural adults living in the southwestern coastal Yunlin County. Specifically, a higher proportion of the elderly live around the western coastal areas in Taiwan [3], and Yunlin County has the second-highest population of the older people (20% are aged >65years old). The county also has a lower population density among the 21 counties of Taiwan. The research team collaborated with a local hospital and five township heads, holding an annual community health screening on Mondays and Tuesdays for 30weeks between March and December 2019. The inclusion criteria were (1) fully independent in daily activities and able to walk to the community center; (2) age>20years and able to communicate in Mandarin or Taiwanese; and (3) agreed to participate in this study and signed the informed consent form. The exclusion criteria were the inability to answer questions or incomplete data.

### Procedure and ethical considerations

This study was approved by Chang Gung Medical Foundation (IRB 201900222A3). The township heads sent messages regarding the free health check-ups and invited individuals to participate in this study. The research team described the study procedures (such as collecting blood samples after 8h of overnight fasting to examine blood sugar and cholesterol levels and the lung function test) to all the participants. Informed consent was obtained from all the participants before this project. Content validity of the health-related behaviors questionnaire was judged to be good (CVI=0.900.92) by a 5 expert panel comprising health education faculty members, metabolic physicians, and nursing faculty members teaching health promotion. Some items within the instruments were revised according to the experts suggestions. Six research assistants were trained for 4h by the investigators. These research assistants were senior nursing students in a Post-RN Bachelor of Nursing Degree Program who held a registered nursing license and received 2 consequent training programs. In session 1, we focused on understanding research background and practicing interview skills. In session 2, 6 research assistants were grouped into 3 pairs to pretest and be familiar with all the questionnaire items. Finally, research assistants were divided into 2 groups to interview each adult, and a 90% accuracy rate of inter-rater reliability was confirmed among the 3 pairs.

### Measurements

1. Demographic characteristics included sex, age, educational level (number of received education years), and self-reported occupation (had a job or not).
2. Cardiometabolic risk factors and MetS were based on the national standard [1, 4]. They included the presence of one of the following five biomarkers: (1) waist circumference (WC)90cm in males, 80cm in females (also known as central obesity), and the WC was measured between the last rib margin and the iliac crest (i.e., the mid-abdominal distance), and by trained senior nursing students; (2) systolic/diastolic blood pressure > 130/85mmHg; (3) HDL-C < 40mg/dL in males, < 50mg/dL in the females; (4) HbA1C > 5.6% was used instead of the blood glucose level, since fasting 8h is not compliant for rural adults in the community screening, and (5) TG level > 150mg/dL. An individual with three or more of these risk factors was classified as having MetS [1].
3. The same certified and experienced technician performed the lung function test using an automated flow-sensing spirometer (Pony Fx-EN13485 is the new generation desktop developed by COSMED). Based on the American Thoracic Society recommendations [24], a dry rolling-seal spirometer, calibrated by using 2 or 3L of precision syringe daily was performed. At least five and up to a maximum of six forced expiratory maneuvers were performed to meet the standards. Three indices of lung function were measured: (1) FVC; (2) predicted FVC value (%); and (3) FEV1/FVC ratio (%). Normal lung function was defined as FEV_1_/FVC70% and FVC80%. Obstructive lung impairment was defined as FEV1/FVC ratio<70% and the predicted FVC value >80%. Restrictive lung impairment was defined as the predicted FVC value <80% and FEV1/FVC>70%. Mixed lung impairment was defined as FEV1/FVC<70% and the predicted FVC value <80% [25]. Spirometer measurement results are derived from the reference values calculated based on the participants age, height, gender, and race, for further mutual comparison and evaluation.
4. Health-related behaviors: According to previous studies and official recommendations for cardiometabolic risk management, three substances and four dietary and exercise behaviors were assessed [1, 26, 27]. Participants were asked: (1) *Do you smoke cigarettes?* non-smokers had never smoked; current/former smokers were current smokers or were previous smokers who had ceased smoking; (2) *Do you chew betel nut?* Participants were classified as non-users if they reported having never chewed, current/former users if they were current users or were previous users and had ceased chewing; (3) *Do you regularly consume alcohol or related beverage?* Participants were classified as non-users if they reported having never drunk, regular users if they reported that they regularly drank at least three times a week; (4) *How often do you consume three portions of vegetables (1.5 bowls) per day*?; (5) *How often do you have two portions of fruit per day?;* (6) *How often do you have 1500 mL of water per day?*; (7) *How often do you have at least exercise for > 30 min, three times per week*?. Participants responses were categorized as never, seldom, usually, or always. For the evaluation, four responses were categorized into two-level frequencies: never/seldom and usually/always. For accuracy and clarity, on drinking measures, participants were shown standardized containers as an example of a bowel/bottle/cup; for example, one bowel or cup contains 240mL of water, and a bottle, 600mL of water.

### Data analysis

The study participants demographics and other characteristics with and without lung function impairment were compared using an independent sample t-test for continuous variables or a chi-square test for categorical variables. To investigate the association between MetS and lung function impairment, we performed multivariable logistic regression analyses adjusted for demographics and other characteristics. All tests were 2-tailed and *P*<0.05 was considered statistically significant. We further conducted post hoc analysis on male subjects with the level of statistical significance set as *P*<0.025. Data analyses were conducted using IBM SPSS Statistics for Windows, version 25 (IBM Corp., Armonk, NY, USA).

## Results

### Demographic characteristics, MetS, and lung function test

**Fig. 1:**
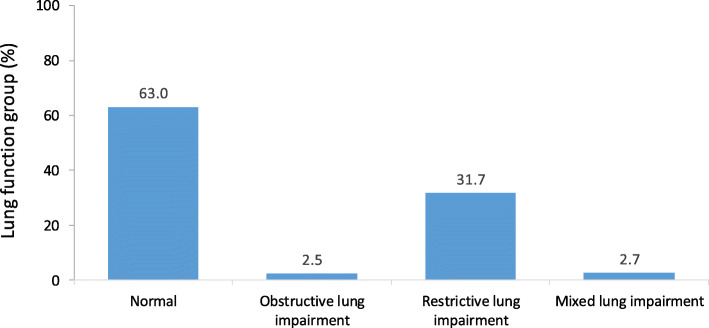
The distribution of lung function group

Excluding 127 individuals with incomplete data, 1653 adults with complete data were analyzed. Of these, 37% (*n*=611) were classified as having lung impairment, including 31.7% restrictive, 2.5% obstructive, and 2.7% mixed type (Fig.1). Among the lung impairment group, 85.8% (*n*=524) had the restrictive type, while others had obstructive type (*n*=42) or mixed type (*n*=45). The low obstructive lung impairment group occurrence did not show any statistical significance. This study was female predominant, 71.6% received less than 9years of education, and 29.9% were illiterates (zero schoolings). The average age was 63.4years, more than half were 65years or older, and the median age was 66years. Eighteen percent of the participants smoked and most of them were males (*p*<0.001), 11.4% consumed alcohol, and 9.8% chewed betel nuts. Overall, 51.5% of the adults had MetS with a mean number of 2.6 cardiometabolic risk factors in the entire sample (Table1).

**Table 1:** Demographics and characteristics of the study subjects according to the lung function test (*N*=1653)

Variable	Total (*n*=1653)	Lung impairment (*n*=611)	Normal (*n*=1042)	*P*
Demographics				
Male sex	598 (36.2)	249 (40.8)	349 (33.5)	0.003
Age, years	63.414.8	68.712.5	60.415.3	<0.001
Age, years				<0.001
2040	166 (10.0)	21 (3.4)	145 (13.9)	
4164	593 (35.9)	171 (28.0)	422 (40.5)	
65	894 (54.1)	419 (68.6)	475 (45.6)	
Education level, years	6.85.5	5.24.9	7.85.5	<0.001
Had a job	783 (47.4)	253 (41.4)	530 (50.9)	<0.001
Substance use (current user)				
Smoking*	300 (18.1)	136 (22.3)	164 (15.7)	0.001
Betel nut chewing	162 (9.8)	82 (13.4)	80 (7.7)	<0.001
Alcoholic drinking	188 (11.4)	77 (12.6)	111 (10.7)	0.228
Lung function				
Forced vital capacity, (L)	2.30.8	1.80.6	2.60.7	<0.001
FVC % of predicted value, %	84.617.6	67.613.6	94.610.6	<0.001
FEV1/FVC ratio, %	83.519.2	83.630.8	83.45.6	0.834
Health-related behaviors (usually/always)				
Intake vegetable 3 portions per day	1119 (67.7)	381 (62.4)	738 (70.8)	<0.001
Fruit >1.5 servings per day	929 (56.2)	303 (49.6)	626 (60.1)	<0.001
Intake of water >1500cc per day	979 (59.2)	341 (55.8)	638 (61.2)	0.030
Exercise >30min, three times per week	501 (30.3)	171 (28.0)	330 (31.7)	0.116
Cardiometabolic risk factors				
Central obesity (WC) ^1^	903 (54.6)	366 (59.9)	537 (51.5)	0.001
Blood pressure ^2^	1018 (61.6)	385 (63.0)	633 (60.7)	0.361
HDL-C^3^	624 (37.7)	271 (44.4)	353 (33.9)	<0.001
HbA1C^4^	1174 (71.0)	483 (79.1)	691 (66.3)	<0.001
Triglyceride^5^	503 (30.4)	212 (34.7)	291 (27.9)	0.004
Metabolic syndrome (MetS)^6^	851 (51.5)	368 (60.2)	483 (46.4)	<0.001
Number of cardiometabolic risk factors	2.61.4	2.81.3	2.41.4	<0.001

*The ratio of male/female smoker are 275:25, Chi-square test=491.31, *P*<0.001

^1^ WC, waist circumference, male >90cm, female >80cm; ^2^ Blood pressure>130/85mmHg; systolic blood pressure / diastolic blood pressure

^3^ HDL-C, high-density lipoprotein cholesterol, male <40mg/dL, female <50mg/dL; ^4^ glycosylated hemoglobin 5.6%; ^5^ Triglyceride 150mg/dL; ^6^ Metabolic syndromes 3 cardiometabolic risk factors; Data were presented as meanstandard deviation or frequency and percentage

FVC, forced vital capacity; FEV1, forced expiratory volume in first one second

On further comparison of lung impairment with demographic characteristics, the lung function impaired adults were more likely to be males (*p*<0.01), older (*p*<0.001), had lower educational level (*p*<0.001), had no job (p<0.001), cigarette smoker (p<0.01), and betel nuts chewer (*p*<0.001). Adults with lung function impairment had worse lung function test indices (p<0.001) except for the FEV1/FVC ratio. The lung-impaired adults had inadequate dietary behaviors, including less intake of adequate vegetables (*p*<0.001), fruit (p<0.001), and water (*p*<0.05). The prevalence of MetS (p<0.001) as well as the average number of cardiometabolic risk factors (p<0.001) were higher in the lung impaired group than in the normal lung function group (Table 1). Further comparison of demographic characteristics with MetS showed that older adults (p<0.001), less education (p<0.001), without a job (*p*<0.01) tended to have MetS. The table of demographics and MetS was not shown.

### Association between substance use, cardiometabolic risks, and lung impairment

Table2 shows the multivariable-adjusted multinomial logistic regression analysis results. Male sex (odds ratio [OR]=2.63, 95% confidence interval [CI]=1.394.97), older age (OR=1.07, 95% CI=1.041.10), cigarette smoking (OR=2.27, 95% CI=1.144.53), and betel nuts chewing (OR=2.33, 95% CI=1.095.01) were independently associated with a higher risk of the obstructive type of lung impairment. In contrast, besides older age and low educational level, the presence of MetS (OR=1.61, 95% CI=1.292.02) or several cardiometabolic risk factors (OR=1.20, 95% CI=1.101.31) was independently associated with a higher risk of restrictive impairment. However, MetS and the number of cardiometabolic risk factors were not significantly associated with the risk of obstructive impairment, which may be due to the small event number.

**Table 2:** Multivariable-adjusted multinomial logistic regression analysis for factors associated with lung impairment (*N*=1653)

	Metabolic syndrome (MetS) as predictor		No. of component of MetS as predictor	
Variable	Risk of obstructive or mixed type (*n*=87)	Risk of restrictive type (*n*=524)	Risk of obstructive or mixed type (*n*=87)	Risk of restrictive type (*n*=524)
Male sex	2.63 (1.394.97)*	1.18 (0.881.59)	2.61 (1.384.93)*	1.20 (0.891.62)
Age, years	1.07 (1.041.10)*	1.02 (1.011.04)*	1.07 (1.041.10)*	1.02 (1.011.04)*
Education level, years	0.995 (0.941.06)	0.95 (0.920.98)*	0.995 (0.941.06)	0.95 (0.920.98)*
Smoking (current user)*	2.27 (1.144.53)*	1.18 (0.781.78)	2.30 (1.154.58)*	1.16 (0.771.76)
Betel nut (current user)	2.33 (1.095.01)*	1.20 (0.722.01)	2.35 (1.095.04)*	1.19 (0.711.98)
Alcoholic drinking (current user)	0.57 (0.271.22)	0.89 (0.581.37)	0.56 (0.271.20)	0.90 (0.581.37)
Intake vegetable 3 portions per day	0.83 (0.391.77)	0.88 (0.631.23)	0.83 (0.391.77)	0.87 (0.631.22)
Fruit >1.5 servings per day	1.43 (0.692.96)	0.88 (0.641.21)	1.44 (0.692.98)	0.88 (0.641.22)
Intake of water >1500cc per day	1.32 (0.792.20)	0.93 (0.741.18)	1.31 (0.792.19)	0.93 (0.741.18)
Exercise >30min, three times per week	0.69 (0.401.20)	0.89 (0.701.14)	0.69 (0.401.19)	0.89 (0.691.13)
Metabolic syndrome (MetS)	0.82 (0.511.33)	1.61 (1.292.02)*		
Number of cardiometabolic risk factors			0.91 (0.761.09)	1.20 (1.101.31)*

Data were presented as adjusted odds ratio and 95% confidence interval

* indicated *P*<0.05

### Smoking, cardiometabolic risks, and lung impairment among male adults

Adult males tended to have smoking habits (*p*<0.001), as well as associated lung impairment (Table 1). We further conducted the post hoc analysis for adult males. The results suggested similar findings that either the presence of MetS (OR=1.90, 97.5% CI=1.302.93) or a greater number of cardiometabolic risk factors (OR=1.30, 97.5% CI=1.091.54) were both risk factors of restrictive lung impairment, but not for obstructive or mixed type due to the small event number (Table3). In addition to older age (OR=1.06, 97.5% CI=1.021.10), smoking habit tended to have a higher risk (OR=2.34, 97.5% CI=1.045.26) in the obstructive or mixed type of lung impairment, but not for restrictive type due to the small event number.

**Table 3:** Multivariable-adjusted multinomial logistic regression analysis for factors associated with lung impairment in male subjects (*N*=598)

	Metabolic syndrome as predictor		No. of component of MetS as predictor	
Variable	Risk of obstructive or mixed type (*n*=62)	Risk of restrictive type (*n*=187)	Risk of obstructive or mixed type (*n*=62)	Risk of restrictive type (*n*=187)
Age, years	1.06 (1.021.10)*	1.03 (1.011.05)*	1.06 (1.021.10)*	1.03 (1.011.05)*
Education level, years	0.95 (0.871.04)	0.97 (0.911.02)	0.95 (0.871.04)	0.97 (0.911.02)
Smoking (current user)	2.34 (1.045.26)*	1.11 (0.661.88)	2.34 (1.045.25)*	1.10 (0.651.85)
Betel nut chewing (current user)	1.98 (0.814.83)	1.26 (0.672.34)	2.02 (0.834.91)	1.22 (0.652.27)
Alcoholic drinking (current user)	0.66 (0.271.60)	0.87 (0.501.51)	0.65 (0.271.58)	0.89 (0.511.54)
Intake vegetable 3 portions per day	0.92 (0.322.63)	0.63 (0.321.24)	0.93 (0.332.63)	0.64 (0.321.28)
Fruit >1.5 servings per day	1.15 (0.423.18)	1.01 (0.511.99)	1.15 (0.423.18)	0.99 (0.501.96)
Intake of water >1500cc per day	1.26 (0.612.62)	0.93 (0.581.47)	1.26 (0.612.62)	0.94 (0.591.49)
Exercise>30min, three times per week	0.79 (0.361.75)	1.13 (0.701.81)	0.79 (0.351.75)	1.14 (0.711.83)
Metabolic syndrome	1.05 (0.532.06)	1.90 (1.232.93)*		
Number of cardiometabolic risk factors			0.98 (0.761.27)	1.30 (1.091.54)*

Data were presented as adjusted odds ratio and 97.5% confidence interval. MetS, metabolic syndromes

* indicated *P*<0.025

## Discussion

Three key findings emerged from this study. Firstly, compared to other studies, the present finding showed a high prevalence of the restrictive type of impaired lung function and MetS. Secondly, apart from the unmodifiable factors of older age and low educational level, MetS or a greater number of cardiometabolic risk factors was independently associated with the restrictive lung impairment. Third, lung-impaired adults tended to adopt an unhealthy lifestyle, especially male smokers and betel nut users, who tended to have a high risk of obstructive or mixed-type lung impairment.

Except for the non-modifiable factors (age, sex, education) of lung function decline, the present findings showed a high prevalence of participants with MetS (51.5%) and cardiometabolic risk factors, which were significantly associated with lung function impairment especially of the restrictive type. These findings are similar to those of previous studies from Japan [14, 15] and South Korea [13], which indicated that individuals with diabetes or poor glycemic control had a higher risk of restrictive lung impairment than those without diabetes. Among a diabetes population, Kim et al. [13] found that body mass index was independently associated with restrictive pulmonary impairment. In our study, all risk factors of MetS, except for blood pressure, significantly impacted lung function. This agrees with previous studies on obesity and diabetes [13, 14]. Besides recognizing the effect of individual cardiovascular risk factors on lung function, one major aim of this study was to clarify the association between lung function impairment and these risk factors designated as MetS, which is now recognized as a good indicator for health care promotion. The relationship between MetS and impaired lung function has been shown, independent of cigarette smoking in our study. In practice, it would be more comprehensive and easier to give comprehensive health education to the community to promote general health instead of action plans to reduce individual risk. No matter the individual risk factors contributing to lung function impairment, it has been shown that besides cardiovascular events, lung function impairment is another important point for health promotion concern in the population with MetS. Hence, an interdisciplinary approach for lifestyle modification for rural adults with impaired lung function is strongly suggested.

The present findings indicate that a high proportion of rural adults adopt an unhealthy lifestyle, including smoking, betel nut chewing, consumption of inadequate vegetable/fruit and water, and inactivity. Studies from Japan and the United States revealed a healthy lifestyle, including healthy eating, non-smoking, less alcohol consumption, and social support, reduce disability in later life [26, 28]. Further, in Spain, Gutirrez-Carrasquilla et al. [17] found that both adherence to the Mediterranean diet and physical activity practices positively impact pulmonary function in subjects with lung disease. These results suggest that a community health promotion program for middle-aged or older people would yield benefits. Further, the present finding showed that 29.9% of participants were illiterate, much higher than the 13% in the general older population [29]. Therefore, if we plan to promote healthy aging, we need to consider individual-tailored activities for the low socioeconomic population since there are a high proportion of participants with low education and those without a job.

The present finding showed that only 5.2% of participants had obstructive or mixed type lung impairment, and cardiometabolic risk factors were significantly associated with restrictive type lung impairment. The possible reason for these results might be due to the small number of individuals with obstructive or mixed types of lung impairment in our present study. Unlike the study by Kim et al. [13] in South Korea, data from the Korea National Health and Nutrition Examination Survey were analyzed. Compared to the non-diabetes group, having diabetes was associated with the restrictive type of lung function impairment and obstructive impairment. In this study, the smoking rate was 18.1%, and most were male smokers (46%). This percentage was higher than the national level percentage. Compared to the official report from the Taiwan smoking survey [30], the smoking rate for those aged 18years in Taiwan was 13%, which included 23.4% for men and 2.4% for women. The present findings are similar to those of the study by Kim et al. [13], where smoking at any point (smoking history) was an independent risk factor for obstructive pulmonary impairment. Evidence shows that cigarette smoking, even among very light smokers, was associated with lung function impairment [22, 23, 31]. Further studies must investigate the influence of smoking and betel nut chewing cessation among adult males with lung function impairment in the community, especially for obstructive impairment.

Despite the valuable findings in this study, some limitations should be noted. Firstly, due to the lack of information on the history of cardiometabolic diseases, such as hypertension or diabetes medications, the present findings might have underestimated the prevalence of cardiometabolic risks. Besides, considering the inconvenience of fasting overnight for 8h for community residents, we used HbA1c>5.6% instead of plasma blood glucose (>100mg/dL). This could affect the prevalence of cardiometabolic risk factors. Secondly, the smoking habit was self-reported, and no confirmation using urinary nicotine levels was obtained. Finally, nonrandom sampling was applied, and health check-ups for each township were performed only on Mondays and Tuesdays. Thus, it might have resulted in a high number of participants with MetS. As a high proportion of participants in the present finding had low education levels and were unemployed, this might limit these findings generalizability.

## Conclusions

A high prevalence of restrictive lung impairment, cardiometabolic risk factors, and unhealthy lifestyle was found among rural adults. Moreover, impaired lung function was significantly associated with MetS or a greater number of cardiometabolic risk factors. In male adults, smoking was independently associated with obstructive or mixed types of lung impairment. Clinicians and primary healthcare providers could reduce the consequences of cardiometabolic-related diseases by initiating individualized and culture-tailored health promotion programs for rural adults with impaired lung function.

## Data Availability

The individual-level data used and/or analyzed for the current study are available from the corresponding author on request.

## Abbreviations

WC: Waist circumference
HDL-C: High-density lipoprotein cholesterol
HbA1C: Glycosylated hemoglobin
MetS: Metabolic syndrome
FVC: Forced vital capacity
FEV1: Forced expiratory volume in first one second

## References

[1] Health Promotion Administration, HPA. Metabolic syndrome. Available from: https://www.hpa.gov.tw/Pages/List.aspx?nodeid=359. (Accessed March 10, 2020).

[2] Lee C, Pickstone N, Facultad J (2017). The future of community health nursing: The hospital in the home. J Community Health..

[3] Ministry of Health and Welfare, Taiwan. Long term care 2.0. Available from: https://1966.gov.tw/LTC/cp-3636-42415-201.html. (Accessed February 1 2020).

[4] Chatterjee A, Harris SB, Leiter LA (2012). Managing cardiometabolic risk in primary care: Summary of the 2011 consensus statement. Can Fam Physician..

[5] Lin MS, Huang TJ, Lin YC (2019). The association of smoking on cardiometabolic risk among male adults with disability in Taiwan. Eur J Cardiovasc Nur..

[6] Mankowski RT, Aubertin-Leheudre M, Beavers (2015). Sedentary time is associated with the metabolic syndrome in older adults with mobility limitations-The LIFE Study. Expl Gerontol.

[7] Morris RW, Taylor AE, Fluharty ME (2015). Heavier smoking may lead to a relative increase in waist circumference: evidence for a causal relationship from a Mendelian randomization meta-analysis. BMJ Open..

[8] McEvoy JW, Nasir K, DeFilippis AP (2015). Relationship of cigarette smoking with inflammation and subclinical vascular disease: the multi-ethnic study of atherosclerosis. Arterioscler Thromb Vasc Biol..

[9] International Diabetes Federation, IDF. The IDF consensus, worldwide definition of the metabolic syndrome. Available from: https://www.idf.org/e-library/consensus-statements/60-idfconsensus-worldwide-definitionof-the-metabolic-syndrome. (Accessed March 10, 2019).

[10] Benjamin EJ, Muntner P, Alonso A (2019). Heart disease and stroke statistics-2019 Update: A report from the American Heart Association. Circulation..

[11] Patel SA, Winkel M, Ali MK (2015). Cardiovascular mortality associated with 5 leading risk factors: national and state preventable fractions estimated from survey data. Ann Intern Med..

[12] Ranasinghe P, Mathangasinghe Y, Jayawardena R (2017). Prevalence and trends of metabolic syndrome among adults in the Asia-pacific region: a systematic review. BMC Public Health..

[13] Kim HY, Sohn TS, Seok H (2017). Prevalence and risk factors for reduced pulmonary function in diabetic patients: The Korea national health and nutrition examination survey. Korean J Intern Med..

[14] Sonoda N, Morimoto A, Tatsumi Y (2018). A prospective study of the impact of diabetes mellitus on restrictive and obstructive lung function impairment: The Saku study. Metabolism..

[15] Sonoda N, Morimoto A, Tatsumi Y (2018). The association between glycemic control and lung function impairment in individuals with diabetes: the Saku study. Diabetol Int..

[16] Jeon YK, Shin MJ, Kim MH (2015). Low pulmonary function is related with a high risk of sarcopenia in community-dwelling older adults: the Korea national health and nutrition examination survey 20082011. Osteoporos Int..

[17] Gutirrez-Carrasquilla L, Snchez E, Hernndez M (2019). Effects of Mediterranean diet and physical activity on pulmonary function: A cross-sectional analysis in the ILERVAS project. Nutrients..

[18] Smith MP, von Berg A, Berdel D (2016). Physical activity is not associated with spirometric indices in lung-healthy German youth. Eur Respir J..

[19] Barrett KE, Barman SM, Brooks HL, et al. Chapter 34: Introduction to pulmonary structure & mechanics. In Ganong's Review of Medical Physiology. 2019;26e:261-8.

[20] Gao C, Zhang X, Wang D (2018). Reference values for lung function screening in 10- to 81-year-old, healthy, never-smoking residents of Southeast China. Medicine..

[21] Roman MA, Rossiter HB, Casaburi R (2016). Exercise, ageing and the lung. Eur Respir J.

[22] Thomas ET, Guppy M, Straus SE (2019). Rate of normal lung function decline in ageing adults: a systematic review of prospective cohort studies. BMJ Open.

[23] World Health Organization, WHO. Tobacco. Available from: http://www.who.int/topics/tobacco/en/. (Accessed March 10, 2020).

[24] American Thoracic Society (1995). Standardization of spirometry, 1994 update. Am J Respir Crit Care Med..

[25] Evans SE, Scanlon PD (2003). Current practice in pulmonary function testing. Mayo Clin Proc..

[26] Hotta R, Makizako H, Doi T (2018). Healthy behaviors and incidence of disability in community-dwelling elderly. Am J Health Behav..

[27] Wang J, Li CM, Chang CF, Jane SW, Chen MY (2015). Psychometric testing of the geriatric health promotion scale. J Nurs Res..

[28] Jacob ME, Yee LM, Diehr PH (2016). Can a healthy lifestyle compress the disabled period in older adults?. J Am Geriatr Soc..

[29] Ministry of Interior, Department of Statistics, Taiwan. Population of 15 Years and over by educational attainment. Available from: https://www.moi.gov.tw/stat/english/node.aspx?sn=7132. (Accessed January 10, 2021).

[30] Ministry of Health and Welfare, Taiwan. Smoking survey. Available from: http://www.hpa.gov.tw/Pages/List.aspx?nodeid=41 (Accessed January 16, 2021).

[31] Rizzi M, Tarsia P, La Spina T (2016). A new approach to detect early lung functional impairment in very light smokers. Respir Physiol Neurobiol..

